# Protective Effect of *N*-Acetylcysteine Amide on Blast-Induced Increase in Intracranial Pressure in Rats

**DOI:** 10.3389/fneur.2017.00219

**Published:** 2017-06-06

**Authors:** Usmah Kawoos, Richard M. McCarron, Mikulas Chavko

**Affiliations:** ^1^Department of Neurotrauma, Naval Medical Research Center, Silver Spring, MD, United States; ^2^Department of Surgery, Uniformed Services University of the Health Sciences, Walter Reed National Military Medical Center, Bethesda, MD, United States

**Keywords:** blast overpressure, traumatic brain injury, intracranial pressure, oxidative stress, *N*-acetylcysteine amide

## Abstract

Blast-induced traumatic brain injury is associated with acute and possibly chronic elevation of intracranial pressure (ICP). The outcome after TBI is dependent on the progression of complex processes which are mediated by oxidative stress. So far, no effective pharmacological protection against TBI exists. In this study, rats were exposed to a single or repetitive blast overpressure (BOP) at moderate intensities of 72 or 110 kPa in a compressed air-driven shock tube. The degree and duration of the increase in ICP were proportional to the intensity and frequency of the blast exposure(s). In most cases, a single dose of antioxidant *N*-acetylcysteine amide (NACA) (500 mg/kg) administered intravenously 2 h after exposure to BOP significantly attenuated blast-induced increase in ICP. A single dose of NACA was not effective in improving the outcome in the group of animals that were subjected to repetitive blast exposures at 110 kPa on the same day. In this group, two treatments with NACA at 2 and 4 h post-BOP exposure resulted in significant attenuation of elevated ICP. Treatment with NACA prior to BOP exposure completely prevented the elevation of ICP. The findings indicate that oxidative stress plays an important role in blast-induced elevated ICP as treatment with NACA-ameliorated ICP increase, which is frequently related to poor functional recovery after TBI.

## Introduction

Blast injury is a prevalent risk for military personnel in the battlefield. The frequency of blast-induced traumatic brain injury (bTBI) is on the rise with long-lasting repercussions seen among war veterans. The early onset of cerebral edema along with delayed and prolonged vasospasm is characteristic features of bTBI (1). Cerebral edema can be a result of cytotoxic and vasogenic mechanisms leading to an increase in cerebral volume and intracranial pressure (ICP) (2–4). Adequate vasoreactivity of cerebral vessels is a critical compensatory mechanism in preventing or minimizing the alteration in ICP, as cerebral volume increases by adjusting cerebral vascular resistance (5). However, prolonged primary and secondary pathophysiological processes may lead to failure of compensatory regulation of cerebral blood flow (CBF) causing an elevation of ICP. Furthermore, activation of systemic inflammation as a direct response to multiple organ injury can result in compromised blood–brain barrier (BBB) integrity and activation of secondary mechanisms of brain injury resulting in an increase in ICP. Implicated secondary mechanisms of TBI include disturbance of ion homeostasis (6), oxidative stress (7), glutamate excitotoxicity (8, 9), activation of inflammatory and immune responses (9), and mitochondrial dysfunction (10). These neurochemical events induce the release of toxic and proinflammatory molecules and reactive oxygen and nitrogen species, which ultimately lead to lipid peroxidation and protein oxidation, BBB disruption, and cerebral edema and cell death (11). Prolonged intracranial hypertension is believed to be associated with poor outcome after TBI (12). The acute therapeutic management of intracranial trauma aims to avoid the development of secondary lesions by initiating neuroprotective interventions to reduce ICP and optimize cerebral perfusion pressure (CPP) and CBF in order to minimize cerebral ischemia and tissue damage (13). Currently, there is no pharmacotherapy that will unequivocally improve clinical outcomes after TBI despite considerable investment by both the pharmaceutical industry and the US National Institutes of Health (NIH).

Accumulation of reactive oxygen species (ROS) is one of the main causes of secondary injury in TBI, leading to activation of a cascade of toxic reactions resulting in damage to neuronal tissue and the vasculature (14, 15). Glutathione (g-glutamylcysteinylglycine, GSH) is an endogenous antioxidant that acts as a scavenger of ROS and reactive nitrogen species (16). A reduced GSH level was shown to initiate oxidative stress-mediated neuronal loss and increases in the levels of exitotoxic molecules leading to cell death *via* necrosis, apoptosis, and exitotoxicity (17). *N*-acetylcysteine (NAC) is a derivative of the endogenous amino acid l-cysteine that serves as a precursor to GSH and reacts with ROS. Over the past decade, NAC demonstrated positive effects in treating psychiatric and neurological disorders (18). However, detailed systematic reviews and meta-analysis failed to show a consistent effect of NAC in various neurological disorders. This, in part, is due to its limited capability to cross the normal BBB (19). *N*-acetylcysteine amide (NACA) is structurally related to NAC with the exception of an amide side chain substitution that gives the compound a neutral charge and improves hydrophobicity and lipophilicity (17). As a result of this minor modification in the chemical structure, NACA has unique physiochemical and pharmacological properties that lead to enhanced penetration into cells, mitochondria, and the central nervous system. This significantly enhances the efficacy of NACA as compared to NAC.

The secondary injury mechanisms activated after induction of TBI evolve over minutes to days and even months after the initial traumatic insult and result from delayed neurochemical, metabolic, and cellular changes. The delayed activation of detrimental reactions suggests that there is a window of opportunity for therapeutic intervention to prevent progressive tissue damage and improve functional recovery after injury. Due to the antioxidative and anti-inflammatory activities and superior BBB permeability of NACA, it could be a strong therapeutic candidate for protection against TBI. It could be even more protective in bTBI by simultaneously preventing oxidative brain damage and effectively reducing systemic inflammation due to its ability to reduce pulmonary damage. The goals of the present study are to characterize the temporal dynamics of elevation in ICP after bTBI and evaluate the efficacy of NACA in preventing the increase in ICP, which is an important indicator of brain damage.

## Materials and Methods

### Animals and Housing Conditions

The animal protocol used in this study was reviewed and approved by the Walter Reed Army Institute of Research/Naval Medical Research Center Institutional Animal Care and Use Committee in compliance with all applicable Federal regulations governing the protection of animals in research. Male Sprague-Dawley rats (weight = 300–350 g; Taconic Farms, NY, USA) were provided at least 1 week of acclimatization period prior to experimentation. The animals were allowed free access to food and water and were provided a daily schedule of dark–light cycle.

### Blast Injury and Grouping

The animals were exposed to blast overpressure (BOP) in a compressed air-driven blast tube which was previously described (20, 21). The animals were placed inside the blast tube with their head facing the blast wave. The anesthetized animals were held in a restrainer with adjustable straps wrapped around the animal’s head, torso, and hind limbs to minimize any possible movement during exposure to blast. They were randomly assigned to groups (*n* = 6 in each group) based on BOP exposure regimen and timing of treatment with NACA (provided by Dr. Glenn Goldstein of Sentient Lifesciences Inc., New York, NY, USA) or phosphate-buffered saline (PBS). The animals were distributed among seven groups which are described in Table 1. The grouping was based on the combination of intensity (72 ± 2 or 110 ± 2 kPa), repetitiveness (1 or 3) of blast, and intraperitoneal administration of NACA. For repetitive BOP exposures, blasts were separated by either 0.5 or 24 h (3 × 110-3d). All groups, except two, received a single injection of NACA (500 mg/1.5 ml PBS/kg of body weight) 2 h after the first BOP exposure. One group (3 × 110-2i) was administered two injections of NACA (2 and 4 h after the first BOP exposure), and the other group (3 × 110-pre) was pre-treated with NACA 15 min prior to BOP exposure. All NACA treated groups had their corresponding placebo (PBS, 1 ml/kg) treated group except 3 × 110-2i. The same placebo group was used for both the 3 × 110 and 3 × 110-2i groups.

**Table 1:** **Grouping of animals based on number and intensity of blasts, interval between blasts, and administration of *N*-acetylcysteine amide (NACA)**.

Group	Description of blast overpressure (BOP) exposure			NACA administration time after the first BOP (h)
	Number	Intensity (kPa)	Interval (h)	
1 × 72	1	72	–	2
3 × 72	3	72	0.5	2
1 × 110	1	110	–	2
3 × 110	3	110	0.5	2
3 × 110-3d	3	110	24	2
3 × 110-2i	3	110	0.5	2, 4
3 × 110-pre	3	110	0.5	−0.25

### ICP Monitoring

Telemetric monitoring was conducted continuously for 7 days by Millar telemetry system (Millar Instruments Inc., Houston, TX, USA) in unrestrained and freely moving rats equipped with an ICP implant, TRM54P (22). The implant consists of a 25-cm long catheter ending in a solid-state, 2-Fr pressure sensor. The pressure sensor was placed in the right lateral ventricle for measuring ICP. Prior to surgical implantation, the sensor was calibrated using a sphygmomanometer method described in Kawoos et al. (23). The implant was turned on for at least 4 h, placed in wet and dark conditions at 37°C, and placed inside a pressure variable and sealed container. The voltage generated by the Millar sensor in response to a known applied pressure over the range of 0–45 mmHg was recorded and used for calibrating the senor as per the manufacturer guidelines. For these sensors, the difference between mean zero offset before and after implantation in rats over 45 ± 5 days in a group of 7 rats was reported to be 2.1 ± 0.4 mmHg (22). The surgical procedure for placement of the implant is detailed elsewhere (24). Briefly, the system consists of a wireless implant for ICP monitoring and data transmission, a SmartPad for receiving wireless transmission, and PowerLab/LabChart (ADInstruments, Colorado Springs, CO, USA) interface for data acquisition and display. After the surgical procedure, the implant was allowed to stabilize for 1 day, following which the animals were exposed to BOP(s). In a previous study, ICP was monitored *via* telemetry by a device surgically implanted in a group of sham animals. After the initial stabilization period of 1 day, the group showed a stable ICP which was within the normal physiological range (21).

### Data Analysis

The data presentation and analysis were simplified by down sampling the continuous recording of ICP to present the representative data for a particular day. The representative data spanned over 3–5.5 h depending on the nature of any event on a particular day. In general, on the day of NACA/PBS injection the length of data presented is up to 2 h after the drug was administered. For inter-group comparison between NACA and PBS at a given BOP regimen, two-way repeated measures analysis of variance (two-way RM ANOVA) followed by Bonferroni *post hoc* analysis (*p* < 0.05 considered significant) was utilized to determine significance of differences between treatments. In order to determine the effect of BOP and NACA/PBS in a group, a moving window analysis was performed in which each data point was compared with the preceding data point. A one-way ANOVA with Tukey’s multiple comparison test (*p* < 0.05 considered significant) was utilized for the moving window analysis method.

## Results

**Figure 1:**
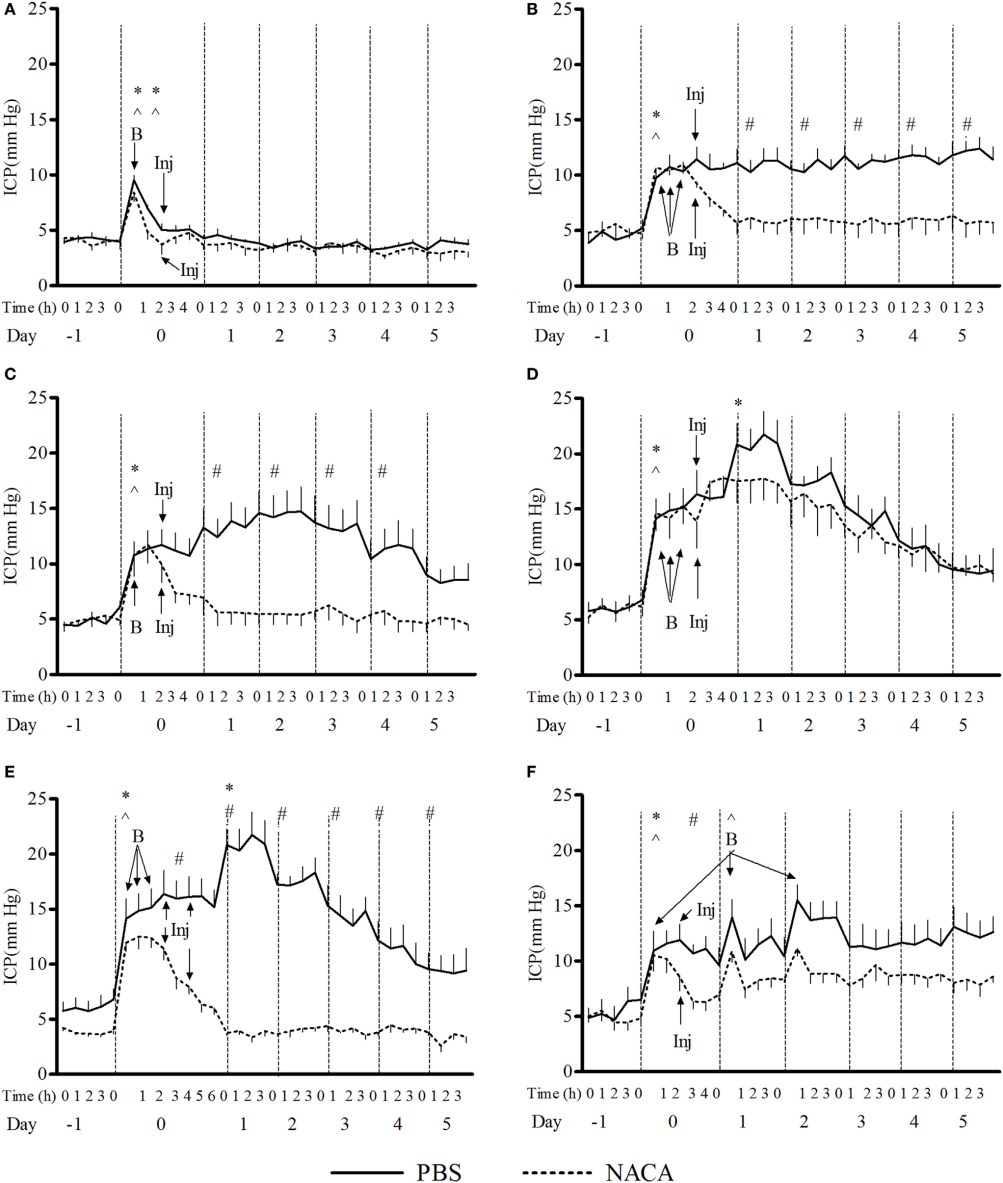
**Changes in intracranial pressure (ICP) with various schedules of blast overpressure (BOP) exposure**. Comparison between *N*-acetylcysteine amide (NACA)- and phosphate-buffered saline (PBS)-treated groups when administered after BOP exposure. **(A)** 1 × 72, **(B)** 3 × 72, **(C)** 1 × 110, **(D)** 3 × 110, **(E)** 3 × 110-2i, and **(F)** 3 × 110-3d. *X*-axis label is not to scale. B, blast; Inj, injection of NACA/PBS. Significant difference in ICP between a given time point and the one preceding it within a group is shown by * in PBS-treated and ^ in NACA-treated groups (one-way ANOVA); and between the two groups is shown by # (two-way RM ANOVA), with *p* < 0.05.

**Figure 2:**
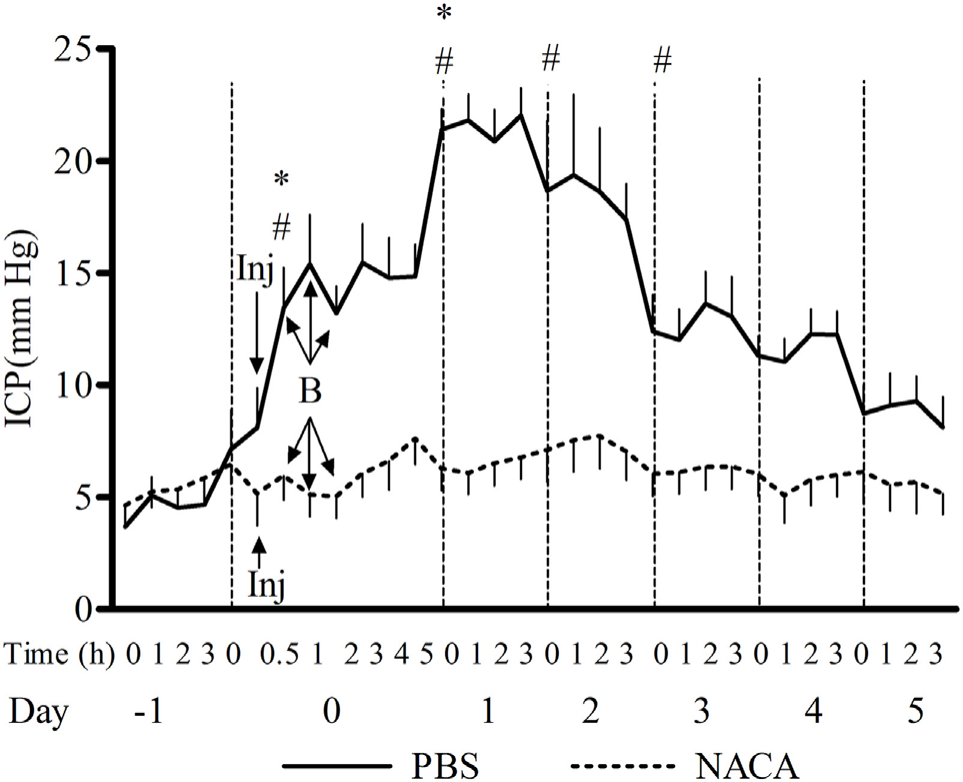
**Changes in intracranial pressure (ICP) after exposure to 3 × 110 kPa blast overpressure (BOP) and the effect of *N*-acetylcysteine amide (NACA) when administered 15 min before the first BOP exposure**. *X*-axis label is not to scale. B, blast; Inj, injection of NACA/phosphate-buffered saline (PBS). Significant difference in ICP between a given time point and the one preceding it within PBS-treated group is shown by * (one-way ANOVA) and between the two groups is shown by # (two-way RM ANOVA), with *p* < 0.05.

The exposure to BOP caused either a transient or prolonged increase in ICP, which was proportional to the number and intensity of BOPs. A single exposure to 72 kPa BOP (Figure 1A) caused a transient increase in ICP on the day of blast, which returned to baseline levels within hours after exposure. Multiple blast exposures at 72 kPa (Figure 1B) led to a gradual and sustained rise in the ICP over time. The pattern of changes in ICP after single (Figure 1C) and repetitive (Figure 1D) BOPs at 110 kPa was distinct from the pattern observed at lower intensity (72 kPa). First, the peak ICP at 110 kPa was greater than that at 72 kPa. Second, the higher intensity groups showed a biphasic response, consisting of an immediate increase in ICP on the day of BOP exposure(s) and a gradual rise to a maximum ICP level. Additionally, there were differences in the time course and amplitude of ICP increase between single and multiple exposures to 110 kPa (Figures 1C,D). The multiple exposure group had a higher peak ICP and took less time to reach the peak (day 1 vs day 2) as compared to the single exposure group. In the 3 × 110-3d group (Figure 1F), ICP increased immediately after each BOP exposure (on days 0, 1, and 2) and declined within hours, though not to the baseline. However ICP remained elevated on days 3, 4, and 5 and did not decrease to baseline levels. A single injection of NACA following exposure to BOP was effective in preventing a significant rise in ICP in 3 × 72 and 1 × 110 groups. There was no significant effect of NACA in the group exposed to three 110 kPa BOP when compared with placebo-treated group (Figure 1D). Therefore, the next group (3 × 110-2i) received two injections of NACA which were administered at 2 and 4 h after the first BOP exposure (Figure 1E). This treatment schedule was sufficient to attenuate the blast-induced increase in ICP to baseline levels on the day of blast without any further increase. In the group exposed to three 110 kPa BOP separated by 24 h (3 × 110-3d), a single dose of NACA reduced the initial increase in ICP in comparison to the placebo-treated group. The effects of NACA treatment were most prominent in the 3 × 110-pre group when the drug was injected prior to the first BOP exposure (Figure 2). Overall, NACA administration had a significant preventive and therapeutic effect on the ICP increase following BOP exposure, with the most significant favorable effects observed in the pre-blast preventive application group.

## Discussion

This study evaluated the efficacy of antioxidant NACA in preventing or attenuating the BOP-induced increase in ICP after exposure to a single or repetitive BOP. Elevated ICP is a marker of complications or mortality after TBI (25), and therefore, the management of ICP is a key approach in improving the outcome in clinical situations. The most serious consequences of increased ICP are reduced CPP and CBF which frequently results in cerebral ischemia (26). The ICP remains within physiological range with small changes in intracranial volume due to compensatory mechanisms by leakage of cerebrospinal fluid and venous blood from the cranium. However, after reaching the critical point in the cerebral pressure–volume relationship, even small changes in the cerebral volume result in a rapid increase in ICP. Further increase in volume can raise ICP until it equals the pressure in cerebral arterioles. At this point, the arterioles may collapse, interrupting blood flow to the brain (26). Increased ICP may also result in excessive compression and herniation of the brain tissue and compression of brain vessels. TBI is a complex process initiated by mechanical trauma to the brain that is followed by imbalance in CBF and cerebral metabolism, activation of oxidative stress, inflammation, edema formation, and apoptosis/necrosis. The neuronal and sub-cellular injury caused by BOP-induced TBI is often times followed by the symptoms of neuropsychiatric disorders (27). Currently, there is no pharmacotherapy that will unequivocally improve clinical outcomes after TBI despite considerable investment by both the pharmaceutical industry and the NIH. Over the last three decades, there have been more than 40 major clinical trials testing neuroprotective agents designed to enhance morphologic and functional recovery following a TBI. All these trials failed in Phase II or Phase III evaluation (28).

Oxidative stress and depletion of endogenous antioxidants are associated with blast-related brain injuries (29–34). The effect of antioxidant treatment in TBI has been extensively reported in several studies (17, 32, 35–43). In animal models of TBI, treatment with free radical scavengers such as superoxide dismutase and OPCV-14117 reduced cerebral infarction and edema (44–46). GSH-promoting compounds such as γ-glutamylcysteine ethyl ester, NAC, and NACA were efficient in neuroprotection in preclinical studies and improved the histological and behavioral outcome after TBI (17, 35, 47, 48). Du et al. (49) reported a marked reduction in the oxidative stress biomarker (4-HNE, end product of lipid peroxidation), c-fos, GFAP, and diffuse axonal injury when rats were treated with a combination of NAC and HPN-07, 1 h after exposure to three blasts at 96.5 kPa (49). NAC was also found to be efficient in reversing behavioral deficits due to brain injury caused by fluid percussion in rats and weight drop in mice (50). In clinical trials, NAC was reported to ameliorate the acute symptoms associated with blast-induced mild TBI in active duty personnel in a combat environment (51). The affected personnel exhibited significant resolution of acute sequelae of mTBI by the seventh day when they were treated with NAC within 24 h after the blast exposure. It was also reported that the time of therapeutic intervention played a key role in the outcome. The chances of symptom resolution in personnel receiving NAC within 24 h after the blast were 86%. These results need to be supported by larger clinical studies with both acute and chronic evaluation. It remains to be investigated whether NAC treatment will be beneficial in treating blast-related moderate or severe TBI.

In addition to positive results, some other studies failed to demonstrate a beneficial effect of NAC on edema and contusion volume after controlled cortical injury (52), and lipid peroxidation in spinal cord injury (53). The conflicting results with NAC may be due to the variability in doses and the type of injury, and its limited bioavailability. At physiological pH, NAC is not readily lipid soluble and carries a negatively charged carboxyl group, which restricts its ability to cross the cellular membranes (54). NACA, an amide derivative of NAC, has a neutralized carboxyl group making it more lipophilic and thus has better permeability through cellular and mitochondrial membranes. This allows NACA to readily replenish GSH levels, scavenge ROS, and attenuate oxidative stress (17). The terminal half-life of reduced NAC in plasma after oral administration (200–400 mg) in adults is 6.25 h (55). With intravenous administration the half-life of NAC in plasma is up to 6 h (56, 57). Due to NACA’s ability to cross the BBB, it was found in brain after oral and intraperitoneal administration, but NAC was not present in the brain (58). NACA may have a longer plasma half-life than NAC when formulated as a co-crystal with an excipient (59). In a model of intracellular oxidation in human red blood cells, NACA reduced oxidative activity of tert-butylhydroxyperoxide (BuOOH) five times more effectively than NAC (60). In the same model, NACA restored 91% of endogenous GSH compared to 15% with NAC suggesting better penetration of NACA through the cellular membrane. NACA was also shown to exhibit a robust anti-inflammatory effect in preventing pulmonary damage after exposure to BOP (61). NACA was shown to confer neuroprotection, improve, and preserve mitochondrial bioenergetics and behavioral and functional outcome after TBI and spinal trauma (40, 41). In a focal penetrating brain injury model, NACA treatment reduced neuronal degeneration and apoptosis and simultaneously increased the levels of manganese superoxide dismutase (mitochondrial antioxidant) (62). The improvement in mitochondrial function and reduction in oxidative stress may inhibit the pathways that lead to inflammatory response after TBI (7).

TBI is a highly complex disorder that is caused by both primary and secondary injury mechanisms. Primary injury mechanisms initiated by over- or underpressurization in reference to the atmospheric pressure (27, 63) result from the transfer of shock wave energy through skull to the brain. The propagation of shock wave through brain causes a non-impact injury also called as “invisible injury” (64). Kucherov et al. suggest that the sub-cellular damage caused by shock waves may be at the scale of nanometer, making it difficult to be detected by conventional modalities. The precise underlying mechanisms of bTBI as well as the neurological, pathophysiological, and functional consequences of bTBI remain unknown. It is still not clear if exposure to BOP or rapid acceleration due to impact is the leading transmission mode of injury. A recent study in a mouse model by Gullotti et al. reported that restricting the movement of head significantly increased survivability and decreased cognitive deficits following blast exposure (65). Head immobilization was most effective against parallel exposure to the blast wave as opposed to a perpendicular orientation. In our study, we reduced linear and rotational head movement by head restriction and exposing animals in frontal orientation to the blast. A detailed literature survey conducted by Song et al. reported 28 research articles on bTBI caused by BOP in the range of 100–200 kPa, showing blast-related injuries to cortex, hippocampus, and cerebellum along with vascular damage and injury to white matter (66). The secondary injury mechanisms evolve over minutes to days and even months after the initial traumatic insult and result from delayed neurochemical, metabolic, and cellular changes. The delayed activation of detrimental reactions suggests that there is a window for therapeutic intervention to prevent progressive tissue damage and improve functional recovery after injury. Treatment with an antioxidant might be most effective when administered at an optimal time after the injury depending on the degree of TBI. In a controlled cortical impact model of TBI, treatment with NAC was effective only when administered 5 min prior to injury or 0.5 or 1 h after the injury (67). In the same study, no effect was seen when the treatment was delayed to 2 h post-injury. After exposure to low intensities of BOP (10, 30, or 60 kPa), ICP increased proportionally with the intensity of exposure, whereas the delay in the maximum elevation of ICP was also proportional to the intensity and repetitiveness of BOP. While the initial elevation took place within 30 min after exposure to 60 kPa, it did not appear until after 2 and 6 h for 30 and 10 kPa, respectively. In all cases, the ICP returned to control levels after 7 days. The ICP increase was accompanied by a decrease in cognitive function of the blast-exposed rats assessed with the Morris water maze (68).

In our study, we observed the relationship between the changes in ICP and the number of exposures to 110 kPa BOP. In comparison with a single exposure, the ICP reached higher peak amplitude and the peak was delayed following multiple exposures to BOP. Animals that were exposed to a single BOP showed maximum ICP on the day after blast and NACA administration reversed the effect of BOP on ICP. However, the repeated exposure group presented a longer delay in reaching the peak ICP levels (2 days after the exposure) and a single dose of NACA was not effective in reversing the effect of BOPs on ICP. In this case, two doses of NACA (separated by 2 h) were found to be adequate in improving the outcome. The effect of NACA against the increase in ICP was more robust in the pre-treated animals indicating that the drug crosses through the BBB even in the situation of non-compromised BBB permeability. In combat settings with a high risk of blast exposure, the military personnel may be pre-emptively treated with NACA before starting the mission. Pre-treatment with NACA might confer neuroprotection against blast-related brain injury in battle fields. However, the adequate dosing of NACA and its toxicity (if any) needs to be fully understood prior to designing clinical trials to assess its efficacy as a neuroprotectant in combat situations. In addition to the direct impact of the shock wave on the skull, some other factors can contribute to the brain damage after exposure to BOP. It has been hypothesized that the shock wave can propagate to the brain *via* blood vessels or cerebral spinal fluid from other parts of the body (69). Air-filled organs such as lungs are the most vulnerable body structures to the primary blast injury (70, 71). Lung compression and activation of the vasovagal reflex was also shown to aggravate brain damage after exposure to BOP. Finally, systemic inflammation in situation of compromised BBB and passage of inflammatory cytokines and chemokines into the brain can contribute to bTBI (72). Exposure to BOP results in contusion or barotrauma-like injury (primary injury) to air-filled organs such as ears, lungs, and the gastrointestinal tract. ROS accumulate in lungs after blunt trauma as a result of reactions catalyzed by iron released from hemoglobin and by inflammatory cells infiltrating the airways. In a study of a rat TBI model, NACA was administered after exposure to a moderate level of blast and the progression of lung inflammation was determined by assessment of inflammatory cytokines and chemokines (61). The data show that NACA significantly ameliorated inflammation in the lungs after 2 days of blast exposure. Myeloperoxidase activity in the lung tissue as an index of neutrophil infiltration was significantly reduced, but not eliminated, by NACA. Similarly, surface CD11b mRNA was significantly decreased but did not return to baseline with NACA treatment. On the other hand, NACA administration completely eliminated the increase in MIP-1, MCP-1, and CINC-1 mRNA 2 days after blast. It could be suggested that the protective effect of NACA on ICP recovery after exposure to BOP may be related to the drug general antioxidative and anti-inflammatory effect in addition to its protective effect against oxidative brain damage.

## Ethics Statement

The animal protocol used in this study was reviewed and approved by the Walter Reed Army Institute of Research/Naval Medical Research Center Institutional Animal Care and Use Committee in compliance with all applicable Federal regulations governing the protection of animals in research.

## Author Contributions

UK made substantial contribution to the conception, design, data acquisition and analysis, drafting and critical revision of the work; final approval of the submitted version; and agreed to be accountable for all aspects of the work. RM and MC made substantial contributions to the conception, design, and critical revision of the work; final approval of the submitted version; and agreed to be accountable for all aspects of the work.

## Disclaimer

The views expressed in this article are those of the authors and do not necessarily reflect the official policy or position of the Department of Navy, Department of Defense, or the U.S. Government. The authors are military service members (or employees of the U.S. Government). This work was prepared as part of the authors’ official duties. Title 17 U.S.C. §105 provides that “Copyright protection under this title is not available for any work of the United States Government.” Title 17 U.S.C. §101 defines a U.S. Government work as a work prepared by military service men or employee of the U.S. Government as part of that person’s official duties.

## Conflict of Interest Statement

The authors declare that the research was conducted in the absence of any commercial or financial relationships that could be construed as a potential conflict of interest.
